# Bilateral compartment syndrome of the lower limbs after urological surgery in the lithotomy position: a clinical case

**DOI:** 10.1590/1677-5449.180117

**Published:** 2019-04-17

**Authors:** José Maciel Caldas dos Reis, Lauro José Mendes Queiroz, Pablo Ferreira Mello, Renan Kleber Costa Teixeira, Fábio de Azevedo Gonçalves

**Affiliations:** 1 Faculdade Metropolitana da Amazônia – FAMAZ, Belém, PA, Brasil.; 2 Hospital do Coração – HCOR, Belém, PA, Brasil.

**Keywords:** compartment syndromes, fasciotomy, postoperative complications, síndromes compartimentais, fasciotomia, complicações pós-operatórias

## Abstract

Acute compartment syndrome of the lower extremities after urological surgery in the lithotomy position is a rare but potentially devastating clinical and medicolegal problem. We report the case of a 67-year-old male who underwent laparoscopic prostatectomy surgery to treat cancer, spending 180 minutes in surgery. Postoperatively, the patient developed acute compartment syndrome of both legs, needing emergency bilateral four-compartment fasciotomies, with repeated returns to the operating room for second-look procedures. The patient also exhibited delayed wound closure. He regained full function within 6 months, returning to unimpaired baseline activity levels. This report aims to highlight the importance of preoperative awareness of this severe complication which, in conjunction with early recognition and immediate surgical management, may mitigate long-term adverse sequelae and improve postoperative outcomes.

## Resumo

A síndrome compartimental aguda dos membros inferiores após cirurgia urológica na posição de litotomia é um problema clínico e médico-legal raro, mas potencialmente devastador. Reportamos o caso de um homem de 67 anos submetido a uma prostatectomia laparoscópica por câncer. A cirurgia durou 180 min. No pós-operatório, o paciente desenvolveu síndrome compartimental aguda de ambos os membros inferiores, necessitando de fasciotomias de urgência, com retornos repetidos à sala de cirurgia para procedimentos adicionais. O paciente também apresentou fechamento tardio da ferida. Ele recuperou a função completa dentro de 6 meses e retornou a um nível de atividade basal irrestrito. Este artigo tem a importância de ressaltar a consciência pré-operatória desta grave complicação que, em conjunto com o reconhecimento precoce e o tratamento cirúrgico imediato, pode mitigar sequelas adversas em longo prazo e melhorar os resultados pós-operatórios.

## INTRODUCTION

The term compartment syndrome refers to a self-propagating cycle that involves the osseous-fascial compartments of the extremities. It occurs when intra-compartmental pressure increases within non-expandable fascia, leading to interstitial edema and increased compartmental pressure, decreasing perfusion of the muscles.^1^^-^ 3 Untreated, it may cause serious complications that can lead to permanent neurological damage, motor dysfunction, acute renal failure, amputations, metabolic acidosis, infections, sepsis, and death.^2^

Lower limb (well leg) compartment syndrome is uncommon and the condition is most often seen after trauma or vascular procedures.^4^ There are few case reports regarding lithotomy positioning and the effects of prolonged surgical procedures with respect to compartment syndrome.^2^^-^ 4 Here, we present a case of bilateral lower limb compartment syndrome after radical laparoscopic prostatectomy surgery, during which the patient spent a prolonged period in the lithotomy position.

## CASE REPORT

A 67-year old man was examined for prostate cancer screening. Prostate biopsy revealed an adenocarcinoma of the prostate, with clinical TNM – PT2cN0M0 stage II; and he was referred to our department for surgical resection. Elective laparoscopic prostatectomy was planned in the Hospital do Coração (Hcor), Belém, PA, Brazil. The patient had been in good health apart from mild hypertension and diabetes. Height, body weight, and BMI were 168 cm, 96.0 kg, and 34.0 kg/m^2^, respectively. The patient was a non-smoker.

The surgery was performed under general anesthesia. The patient wore limb stockings throughout the procedure to maintain temperature. He was placed in the lithotomy position during the procedure using a Levitator© (MIZUHO Corporation, Tokyo, Japan).

The operation was technically difficult, because the patient was overweight, with rich visceral fat and a narrow pelvic cavity. He remained in the lithotomy and head down tilt position for 240 min and the surgery lasted 180 min. His vital signs remained stable throughout. Systolic blood pressure was maintained at a mean of 80 mmHg. During surgery, we did not check the appearance or compression of the lower legs. Postoperative anticoagulation therapy was not administered.

**Figure 1:**
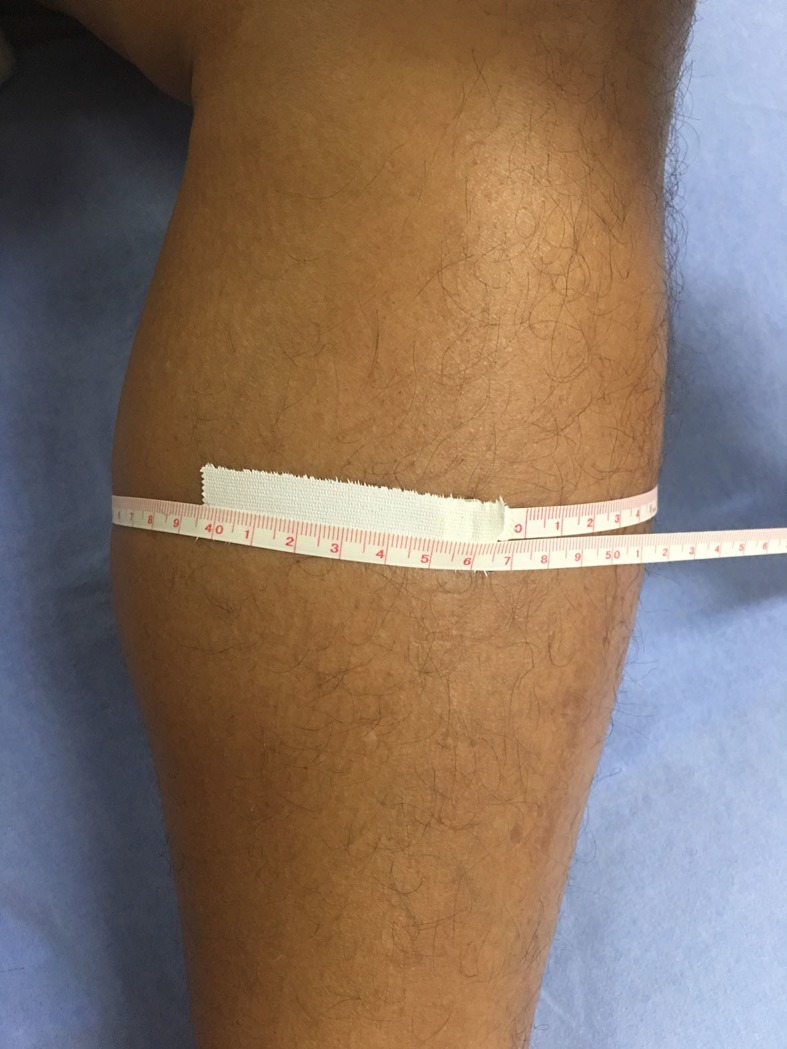
The patient complained of severe cramping-type pain, swelling and considerable tenderness on palpation in both legs.

After the procedure, the patient remained in the intensive care surgical unit. Twelve hours after the procedure, he began to complain of pain and edema in his lower limbs (Figure 1). Femoral, popliteal, and distal pulses were broad and bilaterally symmetrical. Swelling of the calves required attention. An imaging test (Doppler ultrasound) showed no evidence of deep vein thrombosis or occlusion of blood flow. Blood chemistry analysis revealed high levels of lactate dehydrogenase (1830 U/L) and creatine kinase (1240 U/L). Urinalysis results were 3+/3+ for blood and 3+/3+ for protein. Myoglobin was elevated, at 1520 ng/mL.

The patient’s renal function suddenly worsened with concomitant elevation of urea and creatinine. The patient underwent nephrological evaluation and, on the basis of examination findings and laboratory results, catheter hemodialysis was promptly initiated.

The complaints worsened on the second postoperative day. This led us to believe that the patient was suffering from lower limb compartment syndrome. Intracompartmental pressure was not measured.

**Figure 2:**
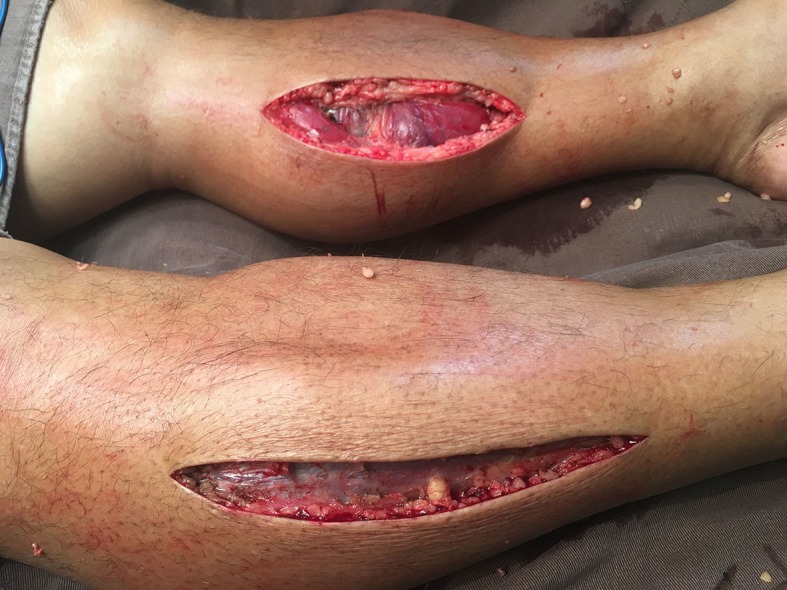
Emergency bilateral fasciotomy with double incisions was performed to release all four compartments.

**Figure 3:**
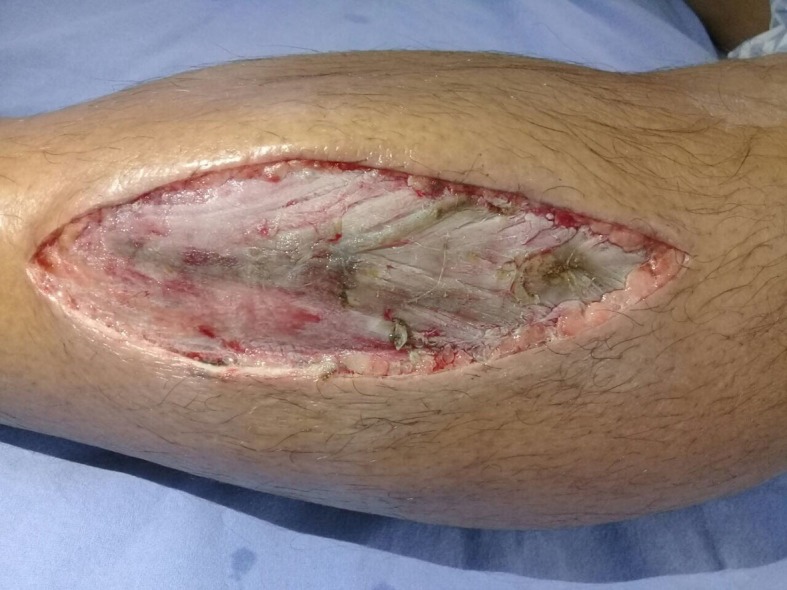
There was evidence of necrosis of the muscles after two days. The incision was left open with sterile dressings.

A bilateral fasciotomy with double incisions was urgently performed to release all four compartments. All compartment muscles were extruded (Figure 2) and anterior lower left limb compartment muscle pain was observed (Figure 3), although there was no muscle necrosis. The incisions were left open with sterile dressings, changed daily.

**Figure 4:**
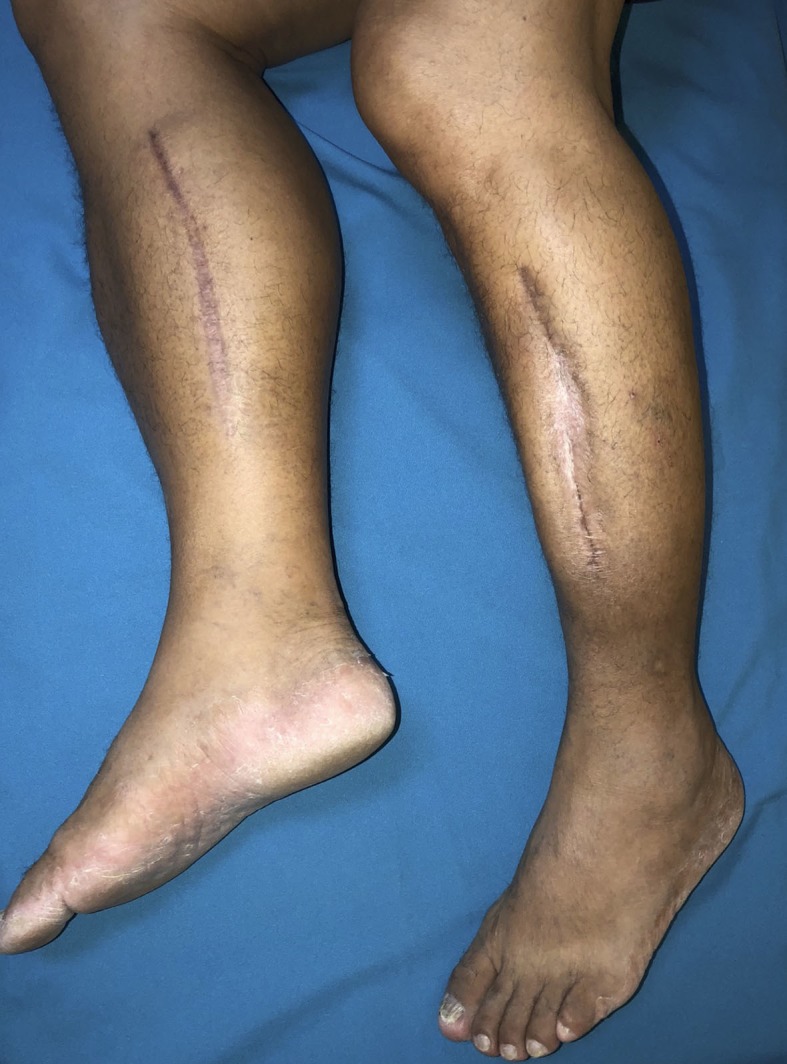
Lower limbs with healed surgical wounds.

Postoperatively, the patient developed a deficit of plantar flexion of the lower limbs, which was worse in the left lower limb. Initially, he was unable to perform dorsiflexion or inversion of either foot. Three days after fasciotomy, he began physiotherapy-led rehabilitation. Closure was performed two weeks later. On the 30th day after the operation, he began to walk with crutches. His condition resolved almost completely, with the exception of discrete motor disorder and pain in the left lower limb. At follow-up, six months after fasciotomy, he reported no motor or sensory impairment and had no complaints of pain (Figure 4).

Written informed consent was obtained from the patient for the publication of this case report. The patient's anonymity has been preserved.

## DISCUSSION

The overall incidence of well leg compartment syndrome in patients after major pelvic surgery in the lithotomy position is estimated to be around 1 in 3,500 cases.^5^ However, the recent literature shows that compartment syndrome might be more common than is generally thought, because the estimated incidence after cystectomy is one in 500.^6^

The physiopathology of lower limb compartment syndrome related to the lithotomy position is not obvious, and the term ‘well leg syndrome’ has recently been adopted for this situation.^2^^,^ 4 ^,^ 5 The possibilities are: arterial hypoperfusion due to the leg being above the level of the heart; venous obstruction from kinking of the veins at the groin or due to external pressure from the stirrups or intermittent compression cuffs; and an increase in compartment pressure resulting from the weight of the limb in the stirrups or passive plantar flexion of the foot.^3^^,^ 5 ^-^ 7

Halliwell et al. and Pfeffer et al. studied lower limb blood pressure in awake volunteers in various lithotomy positions.^7^ They demonstrated a decrease in lower limb blood flow on adoption of the standard lithotomy position and showed that there was a further decrease in pressure when a head down tilt was added.^7^ Local arteriolar pressure has been shown to decrease by 0.78 mmHg for every cm of ankle elevation above the right atrium.^8^

The main risk factors for development of compartment syndrome are: 1) type of leg holder - ankle blood pressures were low and equivalent in lithotomy with heel and calf support; 2) duration of surgery > 4h; 3) pre-existing peripheral vascular disease 4) body mass index > 25 kg/m^2^; and 5) intraoperative hypotension and/or use of vasoconstrictors.^9^

We present a rare case of bilateral lower limb compartment syndrome that developed as a result of a long surgical procedure, requiring lithotomy positioning. Our patient had some risk factors for peripheral vascular disease, such as diabetes, hypertension, and obesity. Moreover, the radical prostatectomy surgery was technically difficult leading to longer duration of surgery in the lithotomy position,

Rapid recognition and prompt intervention are paramount to alleviate this complication and avoid additional damage such as sensory deficits, paralysis, possible limb amputation, and potential multiple organ insufficiency secondary to compartment syndrome.^3^^,^ 10 ^,^ 11

This case highlights a rare complication that has been previously associated with lithotomy positioning and manifests during the postoperative period as pain in the legs, swelling, paresthesia, and, in rare cases, paresis. However, the sensitivity of individual findings for identification of compartment syndrome is low (13-19%).^2^^,^ 3 ^,^ 11 ^,^ 12 Observation of additional clinical findings can increase the chances of accurately identifying compartment syndrome, as high as 93% with the addition of a third symptom.^3^ Thus, although definitive diagnosis can only be made based on direct measurement of intra-compartmental pressure, clinicians should be acutely aware of the potential for compartment syndrome if one or more of the above symptoms are identified in patients following surgery in the lithotomy position. The literature recommends surgical decompression when compartmental pressure is above 30 mm Hg.^3^^,^ 11 ^-^ 13

An urgent single or dual incision fasciotomy must be considered in order to minimize morbidity and even mortality related to compartment syndrome.^2^^-^ 4 The superficial and deep posterior compartments are decompressed through a medial longitudinal incision located 1–2 cm posterior to the medial edge of the tibia.^3^

All the muscles in the four leg compartments were viable upon clinical examination and electrical stimulation tests; there were no signs of frank muscle necrosis, although in the anterior compartments of both limbs, areas of muscular distress with little response to electrical stimulation were observed. The patient required two additional surgical procedures for debridement of the soft tissues and closure of primary wounds. In the first 6 months after discharge, he recovered total unrestricted function and currently has normal quality of life.

As the majority of laparoscopic urological procedures require the patient to be in a lithotomy position, these patients are at particular risk of developing postoperative compartment syndrome, particularly after prolonged procedures of between 2 to 4 h.^3^^,^ 4 ^,^ 14 ^-^ 16 As a result, there are recommendations to help avoid development of this condition. For instance, ensuring intraoperative repositioning of the legs every 2 h can avoid the buildup of pressure in the popliteal fossa and minimize torsion of the popliteal artery; knee flexion beyond 90° should be avoided; when available, external compression devices should be used; and pressure from the surgical assistant on the patient's hip should be avoided.^3^^,^ 6 ^,^ 14 ^-^ 17

This case report emphasizes the importance of understanding the pathophysiology of well leg syndrome in the lithotomy position. Increasing the level of clinical awareness may help identify compartment syndrome in patients who develop pain in the limbs after laparoscopic procedures involving the lithotomy position. Awareness may help patients in all surgeries which require this position.

## CONCLUSION

Thus, we conclude that surgical procedures in which the patient is placed in the lithotomy position for a prolonged time may involve a potential risk of development of compartment syndrome. Appropriate and early management may minimize the harmful consequences of this entity.
